# Placental Growth Factor Is Secreted by the Human Endometrium and Has Potential Important Functions during Embryo Development and Implantation

**DOI:** 10.1371/journal.pone.0163096

**Published:** 2016-10-06

**Authors:** Natalie K. Binder, Jemma Evans, Lois A. Salamonsen, David K. Gardner, Tu’uhevaha J. Kaitu’u-Lino, Natalie J Hannan

**Affiliations:** 1 Translational Obstetrics Group, Department of Obstetrics and Gynaecology, University of Melbourne, Mercy Hospital for Women, Heidelberg, 3084, Australia; 2 School of BioSciences, University of Melbourne, Parkville, 3010, Australia; 3 Hudson Institute of Medical Research, 27–31 Wright St, Clayton, 3168, Australia; 4 Department of Physiology, Monash University, Clayton, 3168, Australia; 5 Department of Obstetrics and Gynecology, Monash University, Clayton, 3168, Australia; University of Georgia, UNITED STATES

## Abstract

Embryo implantation requires synchronized dialogue between the receptive endometrium and activated blastocyst via locally produced soluble mediators. During the mid-secretory (MS) phase of the menstrual cycle, increased glandular secretion into the uterine lumen provides important mediators that modulate the endometrium and support the conceptus during implantation. Previously we demonstrated the importance of vascular endothelial growth factor (VEGF) in the human uterus, particularly with respect to embryo implantation. In the current study, proteomic analysis of human uterine lavage fluid identified the presence of placental growth factor (PlGF) a homolog of VEGF, that binds the VEGF receptor 1 (VEGFR1). Analysis of immunostaining for PlGF in human endometrial tissue across the menstrual cycle (from both fertile and infertile women) revealed PlGF was predominantly localised to glandular and luminal epithelial cells, with staining in the decidualising stromal cells surrounding the maternal spiral arteries in the secretory phase of the menstrual cycle. Immunoreactive PlGF was also detected in subpopulations of endometrial leukocytes. Functional studies demonstrated that culturing mouse embryos with recombinant human (rh)PlGF enhanced blastocyst cell number and outgrowth. Furthermore, treatment of human endometrial epithelial cells (EEC) with rhPlGF enhanced EEC adhesion. Taken together, these data demonstrate that PlGF is abundant in the human endometrium, and secreted into the uterine lumen where it mediates functional changes in cellular adhesion with important roles in implantation.

## Introduction

The human endometrium is receptive to blastocyst implantation for only ~4 days in the mid-secretory (MS) phase of each menstrual cycle [1]. Deficiencies in blastocyst competency and/or endometrial receptivity can impact infertility through implantation failure and early pregnancy loss, and can influence the outcome of in vitro fertilization (IVF).

In the secretory phase of the human menstrual cycle endometrial glandular and luminal epithelial cells transform from relatively inactive to highly secretory polarized cells that transport or synthesize, and secrete substances which accumulate in the uterine lumen. Aside from being a hallmark characteristic of the secretory phase, these secretions are considered to be important mediators of blastocyst development and endometrial function as well as providing important nutritional factors for the developing fetus during the early stages of implantation and placentation[2–5]. A number of studies have examined the composition of endometrial secretions in certain animal species and throughout the menstrual cycle in humans [6–9]. The importance of histotroph from uterine glands is emphasized in a sheep model in which pregnancy cannot be established when the formation of uterine glands, and thus their accompanying secretions, are inhibited[10]. A variety of growth factors and cytokines secreted by uterine glands from women have been identified[2, 5, 11, 12]. Until recently there was little evidence to confirm that components of these secretions were bioactive within the uterine lumen. Disrupted secretion of individual soluble factors including cytokines, growth factors and proteases from the endometrium into the uterine lumen has been correlated with infertility [9, 12, 13]. Therefore studies examining uterine secretions, their bioactivity and function are fundamental to further understanding the importance of maternal-embryonic communication.

Our previous work demonstrated women with unexplained infertility were shown to have reduced levels of vascular endothelial growth factor A (VEGFA) in uterine fluid[11] and a non-vascular role of endometrial derived VEGFA with functional actions on both the maternal surface epithelium and on the peri-implantation blastocyst [11, 14]. In addition VEGF121 and 165 isoforms are predominant in the human uterine lumen while supplementing embryo culture media with VEGF, VEGF121 and VEGF165 had beneficial effects on post-compaction mouse embryo development, outgrowth, implantation and fetal development[14].

Using proteomic applications, the current study determined that placental growth factor (PlGF) a VEGF homolog, is present in human uterine fluid. While PlGF has previously been observed in endometrial tissue[15], it was not previously known to be present in human uterine fluid. In this study the localisation of PlGF in human endometrium across the menstrual cycle and potential roles of PlGF on the peri-implantation embryo and the endometrial epithelium are demonstrated. PlGF had actions on blastocyst cell number and outgrowth as well as endometrial epithelial alterations. Importantly, PlGF has substantial effects on both endometrial epithelial adhesive capacity and on blastocyst outgrowth.

## Materials and Methods

### Ethical approval

Ethical approval was obtained from Monash Surgical Private Hospital; Human Research Ethics Committee (Project No. 04056) and Southern Health Human Research Ethics Committee (Project No. 03066B) for all human sample collections. Written informed consent was obtained from all subjects prior to sample collection. Ethical approval was obtained from The University of Melbourne Animal Ethics Committee (Project ID: 0811074.2) prior to experimentation. All animal experimentation was conducted in accord with accepted standards of humane animal care, as outlined in the Ethical Guidelines of the National Health and Medical Research Council. Mice were sourced from the Zoology Animal Facility (University of Melbourne). Mice were housed in ventilated caging, 3–4 mice/cage. Sawdust was provided for bedding and was replaced on a weekly basis. Animals are on a 12 hour light/dark cycle, at a room temperature (18–22 C; 50% relative humidity). Mice had food available ad libitum as pellets and unrestricted drinking water access.

### Sample collection and patient details

Human uterine lavage fluid (n = 6/pooled) was collected from the secretory phase (days 19–25) of the menstrual cycle. Endometrial biopsies (n = 5-7/cycle phase) were obtained from women with proven fertility (undergoing tubal ligation) and from women with unexplained infertility during the proliferative phase (days 8–11), early secretory phase (days 14–19), mid-secretory phase (days 20–23) and late-secretory phase (days 24–27) of the menstrual cycle (undergoing hysteroscopy, dilatation and curettage).

Menstrual cycle stage was confirmed by histological dating of endometrial tissue, according to the criteria of Noyes *et al*. [16] Patients with uterine abnormalities, such as endometrial polyps, fibroids, endometriosis and endometritis, or who had received steroid hormone therapy in the last 6 months were excluded from the study. Uterine lavage fluid was collected as previously described [5, 11, 17, 18]. Prior to hysteroscopy 3mls of sterile saline was gently infused into the uterine cavity through a fine flexible catheter for a few seconds; the saline solution was then slowly aspirated and centrifuged to remove contaminating cellular debris (including leukocytes, red blood cells and mucous) and stored at -80°C as 0.5ml aliquots.

### Identification of PlGF in human uterine fluid by proteomic analysis

Uterine fluid was collected from the mid-secretory phase of the menstrual cycle and pooled (n = 6). Proteins were extracted from uterine lavage fluid, diluted with 100mM ammonium bicarbonate (Sigma) and reduced with 5mM DTT (Sigma) at 56°C for 30 mins. Samples were incubated with 50 mM ammonium bicarbonate and 25 mM of iodoacetamide (Sigma) for 30 mins at room temperature to alkylate thiol groups. Trypsin (protein: trypsin ratio, 50:1, Promega, Madison, WI) was added and samples digested overnight at 37°C in a humidified chamber. The peptide mixture was fractionated by nanoflow reversed-phase liquid chromatography using a 1200 series Capillary HPLC (Agilent Technologies, Santa Clara CA) online equipped with a nanoAcquity C18 150 mm °ø0.15 mm I.D. column (Waters, Milford,MA). Fractionation was performed at a flow rate of 0.5 μL/min at 45°C in a linear 60-min gradient from 100% solvent A (0.1% formic acid) to 100% solvent B (0.1% Formic acid, 60%acetonitrile). Mass spectra were acquired using an LTQ mass spectrometer equipped with a nanoelectrospray ion source (Thermo Fisher Scientific) for automated MS/MS. Data dependent MS analysis was performed by acquiring one FTMS scan followed by MS2 on the top five most intense ions. Dynamic exclusion was enabled at repeat count 1, exclusion list size 500, exclusion duration 180s, and exclusion mass width 1.5 m/z. Collision induced dissociation was performed by setting the ion isolation width at 2 m/z, normalized collision energy at 35%, activation Q at 0.25 and an activation time at 30 ms.

Resulting spectra were exported in mascot generic file format (.mgf) and analyzed using combinations of UniProtKB_SwissProt (release version 13.3), Uniprot_SwissProt (release version 13.3), UniProtKB_TrEMBL (release version 40.3) and ipi.HUMAN (release version 3.59) using Phenyx (Geneva Bioinformatics, Switzerland, http://phenyx.vital-it.ch/docs/pwi/PWITOC.html). Standard search parameters included a peptide mass tolerance of 1.5 Da, peptide fragment tolerance of 0.8 Da, peptide charge of +2 or +3 and up to 1 missed cleavage allowed. Reverse databases were searched to calculate false discovery rates.

### Endometrial localization of placental growth factor (PlGF)

To validate the endometrium as the source of PlGF identified within uterine lavage fluid the cellular localization of PlGF In endometrial tissues was examined. Immunohistochemical localization was performed using a goat polyclonal antibody raised against human PlGF (PlGF (C-20): sc-1880; Santa Cruz Biotechnologies, USA). Endometrial sections from the proliferative, early secretory, mid-secretory and late secretory phase from both fertile (n = 6-7/phase) and infertile (n = 5-7/phase) women were assessed. Tissue sections were dewaxed and rehydrated prior to mild tryptic digestion at 37°C. Endogenous hydrogen peroxidase activity was blocked with 3% hydrogen peroxide (H_2_O_2_) in dH_2_O for 10 mins at room temperature (RT). Nonspecific binding was blocked with a non-immune blocking solution (10% normal horse serum (NHS; H0146, Sigma), 2% normal human serum (‘in-house’) in TBS/0.1% Tween 20 (Bio-Rad)) in a humidified chamber for 30 mins at RT. Sections were incubated overnight at 4°C with anti-PlGF antibody at a concentration of 1μg/ml in non-immune block. Negative controls were included for each tissue section, where the primary antibody was pre-absorbed with rhPlGF (R&D systems; Minneapolis, MN, USA) at 4°C, for 48 h and applied at 1μg/ml in non-immune block. Biotinylated horse anti-goat IgG (Vector Laboratories, Burlingame, CA, USA) was applied Avidin/biotin conjugated with horseradish peroxidase (ABC-HRP) (Vectastain ABC kit) was applied to sections, followed by application of the peroxidase substrate 3, 3’ diaminobenzidine (DAB) (Dako). Sections were counterstained with Harris Haematoxylin, then dehydrated and mounted. PlGF localization was examined under an Olympus CH30 microscope at various magnifications and high-resolution images were captured.

### Animals and hormonal stimulation

Four-week old Swiss female mice were superovulated with intraperitoneal injections of 5 IU pregnant mare’s serum gonadotrophin (Folligon; Intevet, UK) followed 48 h later by 5 IU hCG (Chorulon; Intervet). Females were mated with Swiss males overnight. The presence of a vaginal plug the following morning was used as an indicator of successful mating.

### Embryo collection and culture

Female mice were euthanized by cervical dislocation. Reproductive tracts were immediately dissected. Pronucleate oocytes were collected approximately 21–22 h post hCG in G-MOPS (Vitrolife) embryo handling medium[19] supplemented with 5 mg/ml human serum albumin (HSA) (Vitrolife, Sweden), followed by cumulus removal in G-MOPS containing 300 IU/ml hyaluronidase (bovine testes, type IV; Sigma Aldrich). Pronucleate oocytes were removed from the hyaluronidase immediately once the cumulus cells had detached, washed twice in G-MOPS and then once in G1 medium (Vitrolife) [20] before culture. Pronucleate oocytes were then combined and randomly assigned to different treatment groups. Embryos were cultured in 20μl drops of G1 medium supplemented with 5 mg/ml HSA under paraffin oil (Ovoil; Vitrolife) at 37°C in 6% CO_2_, 5% O2, 89% N_2_. After 48 h, all embryos were transferred into G2 medium[21] supplemented with 5 mg/ml HSA under the same gas phase conditions for a further 30 hours. Embryos were randomly allocated to one of four treatment groups to examine a dose response of PlGF. (Group 1) Control; G2 media [20] with 5 mg/ml HSA; (Group 2) G2 media containing 5ng/mL of recombinant human (rh)PlGF (R&D systems; Minneapolis, MN, USA); (Group 3) G2 media containing 50ng/mL rhPlGF and (Group 4) G2 media containing 500ng/mL rhPlGF. Embryos were cultured under paraffin oil (Ovoil) at 37°C in 6% CO_2_, 5% O2, 89% N_2_ to the blastocyst stage.

### Blastocyst staining

Blastocyst cell number was determined following final morphological assessment at ~114 h post-hCG (day 5) [11, 14, 22]. Blastocysts (n = 30-39/treatment group) were stained in a solution of 0.1 mg/ml Bisbenzimide (Hoechst, 33342; Sigma Chemical Co.) in 10% ethanol, for 1h at 37°C, and rinsed in G-MOPS with 5 mg/ml HSA and then mounted in glycerol under cover slips on glass slides before being observed under fluorescent light (Nikon TS100-F), and cell numbers counted manually.

### Embryo outgrowth

In order to quantitate blastocyst outgrowth flat bottomed 96-well tissue culture dishes (BD Biosciences, USA) were coated with fibronectin (Fn) (10 μg/ml) (BD Biosciences), rinsed twice with sterile PBS, and incubated with 4mg/ml bovine serum albumin (Sigma Diagnostics, St. Louis, USA) [11, 14]. Wells were rinsed and subsequently filled with 150μl of appropriate experimental medium and equilibrated at 37°C under Ovoil for 3 h prior to the addition of blastocysts. Hatched blastocysts (on day 5 of development) that had been pre-cultured in appropriate medium were placed into the coated wells (1 embryo per well) and incubated for 114h. Outgrowth was examined and images were taken at a matching magnification (10x) at sequential times (66, 74, 90, 98 and 114h following transfer to outgrowth plate) during the culture period with an inverted microscope (Eclipse TS100-F; Nikon, Coherent Scientific Pty. Ltd., SA, Australia) equipped with heated stage to ensure the culture dish was kept at 37°C. The extent of outgrowth for each treatment was obtained by measuring the area of outgrowth in each of the images taken across the experiment using NIS Elements BR 3.00, SP7 Laboratory Imaging software (Nikon). The total area of outgrowth was measured by tracing the leading edge of spread; the area was determined as a numerical value (pixel squared). All images were analysed at matching magnification. The average area of outgrowth of 5 individual blastocysts was calculated for each treatment group: 1) Control; 2) PlGF 5ng/ml; 3) PlGF 50ng/ml and 4) PlGF 500ng/ml. Each experiment was repeated three times (n = 15 embryos were assessed for each of the 4 treatment groups across the 3 experiments). Data are expressed as mean outgrowth ± SEM.

### Endometrial epithelial culture

ECC-1 endometrial epithelial cells (gifted by Prof. Bruce Lessey, Centre for Women’s Medicine, Division of Reproduction Endocrinology and Infertility, Greenville Hospital system, Greenville SC), which have been well characterised previously [23–25] and are representative of the endometrial luminal epithelium. ECC-1 cells were routinely maintained in 1:1 DMEM/F12 media containing 10% fetal calf serum and 1% antibiotic/antimycotic (penicillin/streptomycin) under standard cell culture conditions (37°C, 5% CO_2_ in air).

### Endometrial epithelial adhesion assay

ECC-1 cells were seeded at a density of 1x10^6^ in 25cm^2^ culture flasks in conditions indicated above. 24 h post seeding, cell monolayers were washed twice with calcium/magnesium free phosphate buffered saline (PBS) and deprived of serum for at least 18 h. Serum starved monolayers were subsequently treated with 50ng/ml recombinant human (rh) PlGF (R&D systems) or an equivalent volume of 0.1% bovine serum albumin (BSA, vehicle control) for 24 h in serum free media. Real time cell function was determined as detailed below.

xCelligence real time adhesion and proliferation assays are based on the principle of electrical impedance. Alterations in the local ionic environment at the interface between the gold electrode present within the specialized plates and the solution interface caused by cells attaching to the electrode lead to increases in the electrode impedance. As more cells become attached to the electrode, the electrode impedance increases. RTCA xCelligence E-plates (ACEA Biosciences) were coated with fibronectin (5μg/cm^2^) for one hour prior to cell seeding. 100μl of media containing 50ng/ml rhPlGF or BSA was placed into each well and a background reading performed. rhPlGF/BSA treated cells were trypsinized and seeded onto fibronectin coated plates at 2x10^4^ cells per well. Plates were placed into the xCelligence cradle and readings taken every 15 seconds for 4 hours to determine the effect of PlGF treatment on ECC-1 adhesion. All treatments were seeded in quadruplicate wells on each plate and each individual experiment performed 6 times.

### Statistical Analysis

Data were tested for normal distribution, using three normality tests (1) D'Agostino & Pearson omnibus normality test; 2) Shapiro-Wilk normality test and 3) Kolmogorov-Smirnov test with Dallal-Wilkinson-Lillie for P value). After testing for normal distribution, statistical analysis was performed on raw data. **Blastocyst cell counts** were normally distributed and statistically assessed by analysis of variance followed by Dunnett's multiple comparisons test. **Embryo outgrowth** data were not normally distributed and were tested non-parametrically using the Kruskal-Wallis test. **Endometrial epithelial adhesion assays** Cell adhesion data were not normally distributed. Differences between treatments at specific time points were analyzed using the non-parametric Mann-Whitney test. Real time changes in adhesion over 0–4 hours (continuous) were assessed by linear regression to determine the change in slope over time. All statistical analysis was carried out using PRISM version 6.00 for Mac (GraphPad, SanDiego, CA, USA). P<0.05 was considered significant.

## Results

### Placental growth factor is present in human uterine fluid

Placental growth factor was identified in human uterine fluid using proteomic analysis (LC-MS/MS) of uterine lavage from the mid-secretory phase of women with proven fertility (see Table 1). To validate the presence of PlGF in uterine secretions we performed subsequent independent analysis of its cellular source and function.

**Table 1:** Identification of PlGF following analysis of exported spectra, obtained from LC-MS/MS of uterine lavage from the mid-secretory phase.

Protein Name	Accession Number	Sequence ID	Score	MW (kDa)	Amino acid residues	Calculated isoelectric point
Placenta growth factor [Homo sapiens]	NP_002623	gi20149543	104	45	170	5.92

### Placental growth factor localisation in endometrium across the cycle

**Fig 1:**
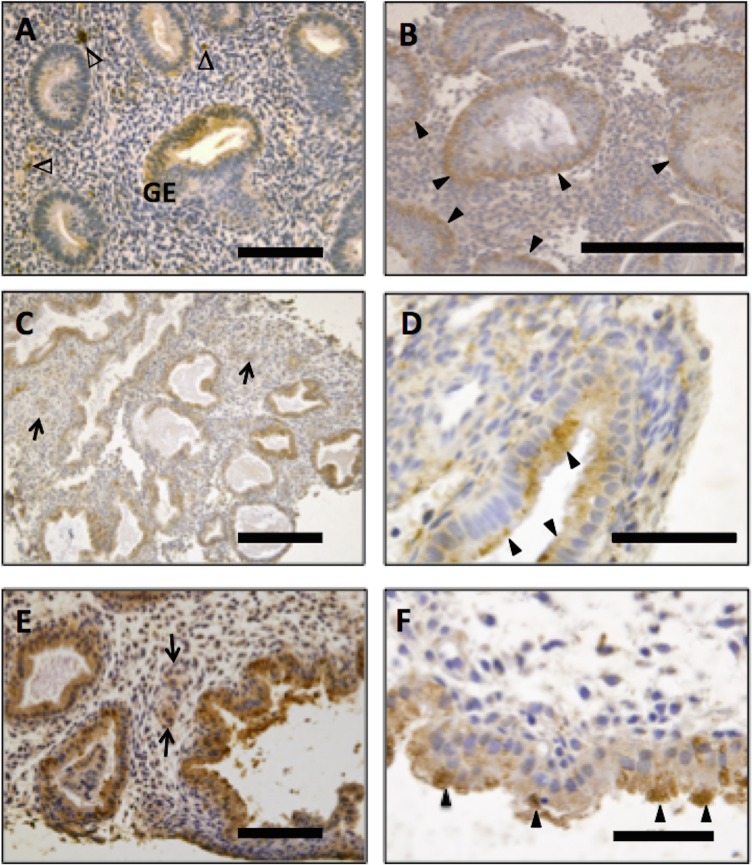
Immunohistochemical localization of Placental Growth Factor (PlGF) in human endometrium. Proliferative phase endometrium showing diffuse weak immunostaining for PlGF in glandular epithelium (GE) and leukocytes (Δ)(A). More intense staining was seen in the early-secretory (B), mid-secretory (C) and late-secretory (D) phases. Immunoreactive staining was localized in basal luminal ‘vesicles’ of the GE in the early-secretory phase (B) these immunoreactive vesicles were apically located in the mid-secretory (D) and late secretory (F) phase, as indicated by arrowheads. Immunoreactive protein was also detected in blood vessels and decidualising stroma surrounding the spiral arterioles (C & E; indicated by arrows). (Endometrial biopsies; n = 5–7 samples/phase of the cycle). No staining was observed in the negative control (isotype matched IgG). Scale bar on A and E = 100 μm, B and C = 200μm, D and F = 50μm.

Positive immunostaining was detected in the human endometrium from all stages of the menstrual cycle, predominantly localized to the glandular and luminal epithelium, with additional immunostaining in decidualised stromal cells, where present, and leukocytes. The intensity and localization of immunostaining varied at different stages of the menstrual cycle. During the proliferative phase, PlGF immunostaining in the glandular epithelium was relatively faint and diffuse with occasional glands staining more intensely (Fig 1A). As the cycle progressed to the early secretory phase, overall immunoreactivity increased and was observed in intensely staining ‘vesicles’ on the basal side of glandular epithelial cells (Fig 1B). During the mid-secretory phase staining intensity in the glands was increased further (Fig 1C) and the subcellular localization of the ‘vesicles’ moved toward the apical surface of the epithelium (Fig 1D). In the late secretory phase, the glandular immunostaining was increased further (Fig 1E), in the apical region of the glandular epithelium of actively secreting glands (Fig 1F). Luminal epithelium was immunoreactive for PlGF during most of the cycle with a marked increase in late secretory phase endometrium, where the staining pattern was similar to that seen in the glandular epithelium (data not shown). Immunoreactive PlGF was also detected in the decidualised stromal cells surrounding the maternal spiral arterioles (Fig 1C and 1E). Both fertile and infertile (unexplained) tissue was assessed, however the overall temporal and spatial immunostaining pattern observed did not differ between the two groups.

### Placental Growth factor enhances blastocyst cell number

**Fig 2:**
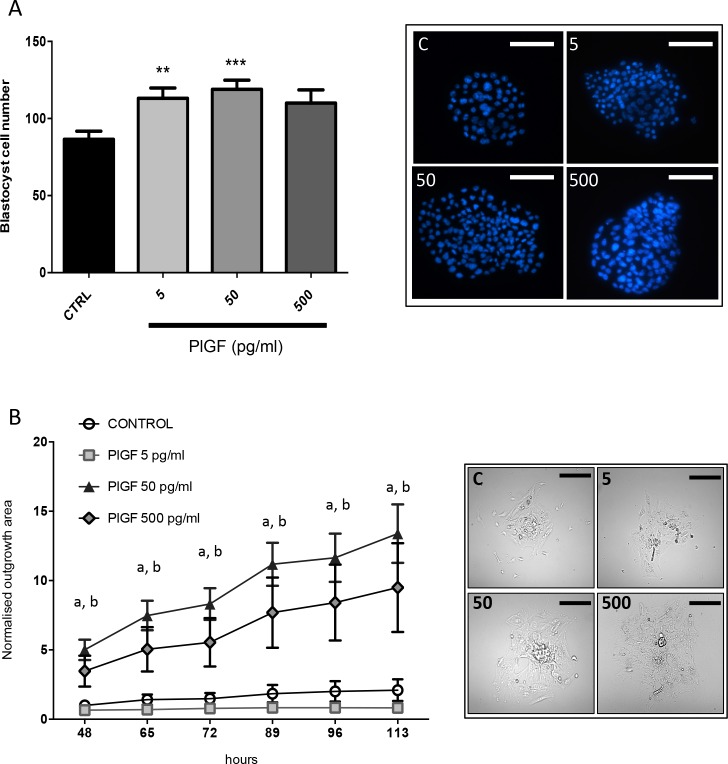
Effects of recombinant human (rh)PlGF on mouse embryo development in culture. Mouse embryos cultured in the presence of 5 and 50ng/mL of rhPlGF had significantly more cells on day 5 of culture than controls. Embryos cultured in the presence of PlGF at a dose of 500ng/mL had no increase in blastocyst cell number. Representative blastocyst images are shown where cell nuclei can be seen (blue) stained with Hoechst. Data are expressed as mean ± SEM. (n = 24-30/treatment group). Data was statistically analyzed by analysis of variance followed by Dunnett's multiple comparisons test (A). Blastocyst outgrowth (on fibronectin 10μg/ml) was significantly enhanced at 46, 65, 72, 89, 96 and 113hrs when embryos were cultured in the presence of rhPlGF (50 and 500 pg/mL) compared to control (B). Blastocysts cultured in PlGF at a dose of 5ng/mL did not exhibit outgrowth on fibronectin. Representative images are shown where blastocyst outgrowth can be visualised (at 113 h) at different doses of PlGF. 5 = 5ng/mL; 50 = 50ng/mL and 500 = 500ng/mL of rhPlGF). (n = 15 embryos/treatment). ‘a’ represents the 50pg/mL dose where p<0.001 and ‘b’ represents the 500pg/mL dose where p<0.01 versus control. Data was tested non-parametrically using Kruskal-Wallis. Scale bar = 100μm.

The number of cells per blastocyst was increased when PlGF was added to the culture media (Fig 2A) compared to control, at doses of 5ng/ml (p< 0.01) and 50ng/ml (p< 0.001). However PlGF at a dose of 500ng/ml had no effect on blastocyst cell number (Fig 2A).

### Placental Growth factor enhances blastocyst outgrowth in vitro

Mouse embryos were used to assess the functional effects of PlGF on blastocyst outgrowth in vitro. An increase in the area of blastocyst outgrowth was observed with rhPlGF treatment at 50ng/ml (p<0.001) and 500ng/ml (p<0.01) across all time points examined versus control (48–113 hours post hatching) compared to control cultured embryos (Fig 2B). However treatment with 5ng/ml PlGF did not alter outgrowth compared to control (Fig 2B).

### Placental Growth factor enhances endometrial epithelial adhesion

**Fig 3:**
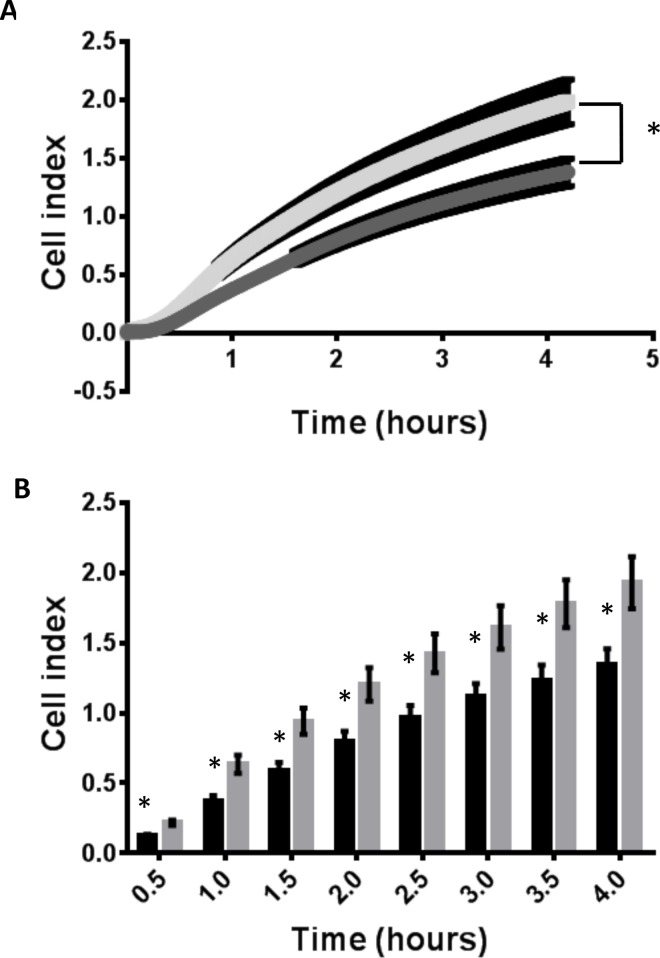
Endometrial epithelial cell adhesion to the extracellular matrix component fibronectin. Treatment with 50ng/ml rhPlGF significantly enhanced adhesion of endometrial epithelial cells to fibronectin in real time (A, p<0.001) across the culture (0–4 hours). Investigation of specific time points demonstrated increased adhesion at 0.5, 1, 1.5, 2, 2.5, 3, 3.5 and 4 hours following treatment with recombinant human PlGF (50ng/ml) versus vehicle control (BSA). Data presented as mean cell index ± SEM. Data representative of n = 6 individual experiments (4 technical replicates within each experiment). *P≤0.01.

Treatment of human endometrial epithelial (ECC-1) cells with rhPlGF (50ng/ml) increased cell adhesion to fibronectin (in real time) over the 0–4 h analysis timeframe (Fig 3A, P<0.001 linear regression). Investigation of specific timepoints demonstrated a rapid 100% increase in adhesion to fibronectin after 0.5 h, with a 46% increase in adhesion maintained to 4 hours (Fig 3B) in comparison to control (BSA; *p≤ 0.01).

## Discussion

Extensive crosstalk between fetal and maternal cells is well recognized as an essential requirement for the establishment of pregnancy particularly during the earliest phase of embryo implantation. The current study identifies that placental growth factor (PlGF), a VEGF homolog, is present in human uterine fluid. Further, this study demonstrates the cellular source of PlGF protein production primarily in luminal and glandular epithelium of human endometrium, with strong staining on the apical surface of the secretory glands during the window of implantation. Functional analysis revealed actions for PlGF on mouse embryo development and outgrowth in vitro; and on endometrial epithelial adhesion to the extracellular matrix fibronectin (abundant at the maternal-fetal interface), supporting a role for secreted PlGF in early endometrial interactions with the blastocyst.

PlGF is a member of the VEGF subfamily, playing key roles in both angiogenesis and vasculogenesis, as well as important roles during embryogenesis. PlGF is mitogenic for endothelial cells[26], enhances chemotaxis in leukocytes[26, 27]. PlGF binds exclusively to the VEGFR1[28] with higher affinity than VEGF[29].

PlGF is highly expressed in placental trophoblast throughout all stages of pregnancy. It has been proposed to control trophoblast growth, differentiation[30, 31] and invasion of the trophoblast into the maternal decidua [32]. PlGF is present in maternal serum during pregnancy: the early dramatic loss of PlGF in the maternal circulation has emerged as a potential predictive marker of preeclampsia[33]. Under normal physiological conditions PlGF expressed at low levels in several other adult organs including the heart, lung, thyroid, skeletal muscle, and adipose tissue [34–36]. Prior to this study however PlGF has not been previously reported to be present in human uterine secretions.

Using LC-MS/MS this study identified PlGF in human uterine fluid. Subsequently PlGF protein was assessed in human endometrium. Immunoreactive protein was abundantly localized to the luminal and glandular epithelium. The staining pattern observed in the secretory phase demonstrates immunoreactive protein in the early secretory phase was predominantly localized to the basal side of the epithelial cells, where as in the later secretory phase PlGF protein was predominantly localized in the apical region of the epithelial cells. Such a staining pattern suggests PlGF is apically secreted from the endometrial epithelium into the uterine lumen, further validating its presence in uterine secretions. Immunoreactive PlGF was also observed in decidual stromal cells, sub-populations of leukocytes and the maternal vasculature. Likewise VEGFR1 is expressed by maternal endothelium and sub-populations of leukocytes[37]. In this setting signaling via the VEGFR1 is thought to play key roles in vascular remodeling associated with early pregnancy. Therefore the co-expression of PlGF and VEGFR1 in the maternal vasculature, as well as increased local production of PlGF in the surrounding decidualising stroma may have functional implications in spiral artery remodeling. Furthermore PlGF has chemotactic actions on leukocytes [27] and may therefore be involved in leukocyte recruitment important for maternal spiral artery remodeling necessary during the establishment of pregnancy[38]. The intensity of PlGF localization within the glandular epithelium appeared to increase as the cycle progressed with peak levels of protein observed during the mid-late secretory phase. There was no obvious difference in protein abundance or localization revealed by immunostaining in tissues from women with unexplained infertility compared to fertile. Here we demonstrate that PlGF is produced by the human endometrium and released into the uterine lumen.

Given that VEGFR1 is expressed by the surface endometrial epithelium[39] we postulated PlGF may have autocrine actions on the luminal and glandular epithelium. Endometrial epithelial cells cultured with rhPlGF displayed a significant increase in endometrial epithelial adhesion on fibronectin, an important ECM component at the forefront of the human maternal-fetal interface [40–42], suggesting an increased capacity for blastocyst adherence and attachment. Regulation of adhesion molecules during implantation is a very important aspect of early embryo implantation in both the human and the mouse [40]. Increased adhesive capacity of the endometrial epithelium is required to facilitate initial attachment and adhesion of the hatched blastocyst to the maternal luminal epithelium[43]. Contact between peri-implantation blastocysts and fibronectin enhances the trophectoderm/trophoblast adhesion to ECM[40, 42, 44]. Thus our findings demonstrating that PlGF enhances human endometrial epithelial adhesion to fibronectin suggests an increased capacity to bind to the peri-implantation embryo. Together this work demonstrates actions of endometrial derived PlGF at the maternal-embryonic interface and specifically of secreted PlGF on embryo development and endometrial receptivity.

Here we demonstrate that PlGF is produced by the human endometrium and released into the uterine lumen, similarly to VEGF[11]. Aside from having its own important actions, PlGF is thought to enhance the response to VEGF by forming VEGF/PlGF heterodimers, which have been found to be more active than PlGF homodimers and nearly as potent as VEGF homodimers in assays of mitogenesis [45, 46].

It is important to note that despite high levels of expression of PlGF in the placenta and roles in placentation, mice lacking PlGF (*plgf-/-* knock out) were reported to be fertile and have normal embryonic development [45]. This is perhaps not surprising given the redundancy of many factors at implantation, which provides a fail-safe mechanism for such an important process. In this case VEGF is likely to be able to bind to the VEGFR1 in the absence of PlGF and therefore signaling through VEGFR1 would remain. Their binding to VEGFR1 and potency as homo and heterodimers requires further consideration in terms of their combined functional roles during implantation.

In summary, the current study demonstrates the PlGF is detectable in the human endometrial glands and in human uterine secretions. The localization and the temporal changes in PlGF localization support a role for PlGF in influencing embryo implantation. Functional analyses suggest PlGF may have roles during the initiation of embryo implantation on both the endometrium and the developing embryo. These findings support the paradigm of paracrine and autocrine dialogue between the blastocyst and endometrial epithelium at the materno-fetal interface during embryo implantation.

## References

[1] Navot D, Bergh P. Preparation of the human endometrium for implantation. Ann N Y Acad Sci. 1991;622:212–9. Epub 1991/01/01. 10.1111/j.1749-6632.1991.tb37864.x . 2064182

[2] Hempstock J, Cindrova-Davies T, Jauniaux E, Burton GJ. Endometrial glands as a source of nutrients, growth factors and cytokines during the first trimester of human pregnancy: a morphological and immunohistochemical study. Reprod Biol Endocrinol. 2004;2:58 10.1186/1477-7827-2-58 . 15265238 PMC493283

[3] Gray CA, Bartol FF, Tarleton BJ, Wiley AA, Johnson GA, Bazer FW, et al Developmental biology of uterine glands. Biol Reprod. 2001;65(5):1311–23. 10.1095/biolreprod65.5.1311 . 11673245

[4] Burton GJ, Watson AL, Hempstock J, Skepper JN, Jauniaux E. Uterine glands provide histiotrophic nutrition for the human fetus during the first trimester of pregnancy. J Clin Endocrinol Metab. 2002;87(6):2954–9. 10.1210/jcem.87.6.8563 . 12050279

[5] Hannan NJ, Stephens AN, Rainczuk A, Hincks C, Rombauts LJ, Salamonsen LA. 2D-DiGE analysis of the human endometrial secretome reveals differences between receptive and nonreceptive states in fertile and infertile women. J Proteome Res. 2010;9(12):6256–64. Epub 2010/10/12. 10.1021/pr1004828 . 20925431

[6] Simon C, Mercader A, Frances A, Gimeno MJ, Polan ML, Remohi J, et al Hormonal regulation of serum and endometrial IL-1 alpha, IL-1 beta and IL-1ra: IL-1 endometrial microenvironment of the human embryo at the apposition phase under physiological and supraphysiological steroid level conditions. J Reprod Immunol. 1996;31(3):165–84. 10.1016/0165-0378(96)00982-5 . 8905550

[7] White FJ, Burghardt RC, Hu J, Joyce MM, Spencer TE, Johnson GA. Secreted phosphoprotein 1 (osteopontin) is expressed by stromal macrophages in cyclic and pregnant endometrium of mice, but is induced by estrogen in luminal epithelium during conceptus attachment for implantation. Reproduction. 2006;132(6):919–29. 10.1530/REP-06-0068 . 17127752

[8] Spencer TE, Bazer FW. Uterine and placental factors regulating conceptus growth in domestic animals. J Anim Sci. 2004;82 E-Suppl:E4-13. . 15471813 10.2527/2004.8213_supplE4x

[9] Mikolajczyk M, Wirstlein P, Skrzypczak J. Leukaemia inhibitory factor and interleukin 11 levels in uterine flushings of infertile patients with endometriosis. Hum Reprod. 2006;21(12):3054–8. 10.1093/humrep/del225 . 17000646

[10] Gray CA, Burghardt RC, Johnson GA, Bazer FW, Spencer TE. Evidence that absence of endometrial gland secretions in uterine gland knockout ewes compromises conceptus survival and elongation. Reproduction. 2002;124(2):289–300. 10.1530/reprod/124.2.289 . 12141942

[11] Hannan NJ, Paiva P, Meehan KL, Rombauts LJ, Gardner DK, Salamonsen LA. Analysis of fertility-related soluble mediators in human uterine fluid identifies VEGF as a key regulator of embryo implantation. Endocrinology. 2011;152(12):4948–56. Epub 2011/10/27. en.2011-1248 [pii] 10.1210/en.2011-1248 . 22028446

[12] Boomsma CM, Kavelaars A, Eijkemans MJ, Amarouchi K, Teklenburg G, Gutknecht D, et al Cytokine profiling in endometrial secretions: a non-invasive window on endometrial receptivity. Reprod Biomed Online. 2009;18(1):85–94. 10.1016/s1472-6483(10)60429-4 . 19146774

[13] Dimitriadis E, Stoikos C, Stafford-Bell M, Clark I, Paiva P, Kovacs G, et al Interleukin-11, IL-11 receptoralpha and leukemia inhibitory factor are dysregulated in endometrium of infertile women with endometriosis during the implantation window. J Reprod Immunol. 2006;69(1):53–64. 10.1016/j.jri.2005.07.004 . 16310857

[14] Binder NK, Evans J, Gardner DK, Salamonsen LA, Hannan NJ. Endometrial signals improve embryo outcome: functional role of vascular endothelial growth factor isoforms on embryo development and implantation in mice. Hum Reprod. 2014;29(10):2278–86. 10.1093/humrep/deu211 . 25124669

[15] Chen X, Jin X, Liu L, Man CW, Huang J, Wang CC, et al Differential expression of vascular endothelial growth factor angiogenic factors in different endometrial compartments in women who have an elevated progesterone level before oocyte retrieval, during in vitro fertilization-embryo transfer treatment. Fertil Steril. 2015 Epub 2015/07/06. 10.1016/j.fertnstert.2015.06.021 . 26143364

[16] Noyes RW, Hertig AT, Rock J. Dating the endometrial biopsy. American journal of obstetrics and gynecology. 1975;122(2):262–3. . 1155504 10.1016/s0002-9378(16)33500-1

[17] Hannan NJ, Nie G, Rainzcuk A, Rombauts LJ, Salamonsen LA. Uterine lavage or aspirate: which view of the intrauterine environment? Reprod Sci. 2012;19(10):1125–32. Epub 2012/05/01. 1933719112443879 [pii] 10.1177/1933719112443879 . 22544848

[18] Hannan NJ, Stoikos CJ, Stephens AN, Salamonsen LA. Depletion of high-abundance serum proteins from human uterine lavages enhances detection of lower-abundance proteins. J Proteome Res. 2009;8(2):1099–103. Epub 2008/12/31. 10.1021/pr800811y [pii]. . 19113883

[19] Lane M, Gardner D.K. Preparation of gametes, in vitro maturation, in vitro fertilization, embryo recovery and transfer In: Gardner DK, Lane M., Watson A.J., editor. A Laboratory Guide to the Mammalian Embryo. New York: Oxford Press,; 2004 p. 24–40.

[20] Gardner DK, Lane M. Embryo culture systems In: Gardner DK, editor. In Vitro Fertilization–A Practical Approach. New York: Informa Healthcare; 2007 p. 221–82.

[21] Gardner DK, Lane M. Mammalian preimplantation embryo culture. Methods Mol Biol. 2014;1092:167–82. Epub 2013/12/10. 10.1007/978-1-60327-292-6_11 . 24318820

[22] Binder NK, Hannan NJ, Gardner DK. Paternal diet-induced obesity retards early mouse embryo development, mitochondrial activity and pregnancy health. PLoS One. 2012;7(12):e52304 Epub 2013/01/10. 10.1371/journal.pone.0052304PONE-D-12-23007 [pii]. . 23300638 PMC3531483

[23] Desai NN, Kennard EA, Kniss DA, Friedman CI. Novel human endometrial cell line promotes blastocyst development. Fertility & Sterility. 1994;61(4):760–6. 10.1016/s0015-0282(16)56659-x 7512055

[24] King AE, Fleming DC, Critchley HO, Kelly RW. Regulation of natural antibiotic expression by inflammatory mediators and mimics of infection in human endometrial epithelial cells. Molecular Human Reproduction. 2002;8(4):341–9. 10.1093/molehr/8.4.341 11912282

[25] Mo B, Vendrov AE, Palomino WA, DuPont BR, Apparao KB, Lessey BA. ECC-1 cells: a well-differentiated steroid-responsive endometrial cell line with characteristics of luminal epithelium. Biol Reprod. 2006;75(3):387–94. 10.1095/biolreprod.106.051870 . 16707768

[26] Carmeliet P. Mechanisms of angiogenesis and arteriogenesis. Nat Med. 2000;6(4):389–95. Epub 2000/03/31. 10.1038/74651 . 10742145

[27] Clauss M, Weich H, Breier G, Knies U, Rockl W, Waltenberger J, et al The vascular endothelial growth factor receptor Flt-1 mediates biological activities. Implications for a functional role of placenta growth factor in monocyte activation and chemotaxis. The Journal of biological chemistry. 1996;271(30):17629–34. Epub 1996/07/26. 10.1074/jbc.271.30.17629 . 8663424

[28] Park JE, Chen HH, Winer J, Houck KA, Ferrara N. Placenta growth factor. Potentiation of vascular endothelial growth factor bioactivity, in vitro and in vivo, and high affinity binding to Flt-1 but not to Flk-1/KDR. The Journal of biological chemistry. 1994;269(41):25646–54. Epub 1994/10/14. . 7929268

[29] Olofsson B, Korpelainen E, Pepper MS, Mandriota SJ, Aase K, Kumar V, et al Vascular endothelial growth factor B (VEGF-B) binds to VEGF receptor-1 and regulates plasminogen activator activity in endothelial cells. Proc Natl Acad Sci U S A. 1998;95(20):11709–14. Epub 1998/09/30. 10.1073/pnas.95.20.11709 9751730 PMC21705

[30] Maglione D, Guerriero V, Rambaldi M, Russo G, Persico MG. Translation of the placenta growth factor mRNA is severely affected by a small open reading frame localized in the 5' untranslated region. Growth factors. 1993;8(2):141–52. Epub 1993/01/01. 10.3109/08977199309046934 . 8466755

[31] Khaliq A, Li XF, Shams M, Sisi P, Acevedo CA, Whittle MJ, et al Localisation of placenta growth factor (PIGF) in human term placenta. Growth factors. 1996;13(3–4):243–50,color plates I-II,pre bk cov. Epub 1996/01/01. 10.3109/08977199609003225 . 8919031

[32] Vuorela P, Hatva E, Lymboussaki A, Kaipainen A, Joukov V, Persico MG, et al Expression of vascular endothelial growth factor and placenta growth factor in human placenta. Biol Reprod. 1997;56(2):489–94. Epub 1997/02/01. 10.1095/biolreprod56.2.489 . 9116151

[33] Levine RJ, Maynard SE, Qian C, Lim KH, England LJ, Yu KF, et al Circulating angiogenic factors and the risk of preeclampsia. The New England journal of medicine. 2004;350(7):672–83. Epub 2004/02/07. 10.1056/NEJMoa031884 . 14764923

[34] Viglietto G, Maglione D, Rambaldi M, Cerutti J, Romano A, Trapasso F, et al Upregulation of vascular endothelial growth factor (VEGF) and downregulation of placenta growth factor (PlGF) associated with malignancy in human thyroid tumors and cell lines. Oncogene. 1995;11(8):1569–79. Epub 1995/10/19. . 7478581

[35] Persico MG, Vincenti V, DiPalma T. Structure, expression and receptor-binding properties of placenta growth factor (PlGF). Current topics in microbiology and immunology. 1999;237:31–40. Epub 1999/01/20. 10.1007/978-3-642-59953-8_2 . 9893344

[36] Voros G, Maquoi E, Demeulemeester D, Clerx N, Collen D, Lijnen HR. Modulation of angiogenesis during adipose tissue development in murine models of obesity. Endocrinology. 2005;146(10):4545–54. Epub 2005/07/16. 10.1210/en.2005-0532 . 16020476

[37] Moller B, Rasmussen C, Lindblom B, Olovsson M. Expression of the angiogenic growth factors VEGF, FGF-2, EGF and their receptors in normal human endometrium during the menstrual cycle. Mol Hum Reprod. 2001;7(1):65–72. Epub 2001/01/03. 10.1093/molehr/7.1.65 . 11134362

[38] Loke YW, King A. Immunological aspects of human implantation. Journal of reproduction and fertility Supplement. 2000;55:83–90. Epub 2000/07/13. . 10889837

[39] Girling JE, Rogers PA. Regulation of endometrial vascular remodelling: role of the vascular endothelial growth factor family and the angiopoietin-TIE signalling system. Reproduction. 2009;138(6):883–93. Epub 2009/09/17. REP-09-0147 [pii] 10.1530/REP-09-0147 . 19755482

[40] Shimomura Y, Ando H, Furugori K, Kajiyama H, Suzuki M, Iwase A, et al Possible involvement of crosstalk cell-adhesion mechanism by endometrial CD26/dipeptidyl peptidase IV and embryonal fibronectin in human blastocyst implantation. Mol Hum Reprod. 2006;12(8):491–5. Epub 2006/04/20. gal019 [pii] 10.1093/molehr/gal019 . 16621928

[41] Aplin J. Maternal influences on placental development. Semin Cell Dev Biol. 2000;11(2):115–25. 10.1006/scdb.2000.0157 . 10873708

[42] Thorsteinsdottir S. Basement membrane and fibronectin matrix are distinct entities in the developing mouse blastocyst. Anat Rec. 1992;232(1):141–9. Epub 1992/01/01. 10.1002/ar.1092320116 . 1536459

[43] Singh H, Aplin JD. Adhesion molecules in endometrial epithelium: tissue integrity and embryo implantation. J Anat. 2009;215(1):3–13. 10.1111/j.1469-7580.2008.01034.x 19453302 PMC2714633

[44] Wang J, Mayernik L, Armant DR. Integrin signaling regulates blastocyst adhesion to fibronectin at implantation: intracellular calcium transients and vesicle trafficking in primary trophoblast cells. Dev Biol. 2002;245(2):270–9. 10.1006/dbio.2002.0644 . 11977980

[45] Carmeliet P, Moons L, Luttun A, Vincenti V, Compernolle V, De Mol M, et al Synergism between vascular endothelial growth factor and placental growth factor contributes to angiogenesis and plasma extravasation in pathological conditions. Nat Med. 2001;7(5):575–83. Epub 2001/05/01. 10.1038/87904 . 11329059

[46] Bottomley MJ, Webb NJ, Watson CJ, Holt L, Bukhari M, Denton J, et al Placenta growth factor (PlGF) induces vascular endothelial growth factor (VEGF) secretion from mononuclear cells and is co-expressed with VEGF in synovial fluid. Clinical and experimental immunology. 2000;119(1):182–8. Epub 1999/12/22. 10.1046/j.1365-2249.2000.01097.x 10606981 PMC1905543

